# A novel mutation in IL36RN underpins childhood pustular dermatosis

**DOI:** 10.1111/jdv.13034

**Published:** 2015-02-16

**Authors:** J.M. Ellingford, G.C.M. Black, T.H. Clayton, M. Judge, C.E.M. Griffiths, R.B. Warren

**Affiliations:** ^1^ Manchester Centre for Genomic MedicineCentral Manchester University Hospitals NHS Foundation TrustManchester Academic Health Science CentreManchesterUK; ^2^ The Dermatology CentreSalford Royal NHS Foundation TrustManchester Academic Health Science CentreUniversity of ManchesterManchesterUK

## Abstract

**Background:**

Chronic pustular dermatoses are severe and debilitating autoinflammatory conditions that can have a monogenic basis. Their clinical features are, however, complex with considerable overlap. Null and missense mutations in the genes encoding interleukin (IL)‐1 family (IL‐1 and IL‐36) anti‐inflammatory receptor antagonist (Ra) cytokines can underlie the development of severe pustular dermatoses.

**Objective:**

We present a clinical and genetic study of four children of Pakistani descent with similar clinical presentations and treatment course, each of whom suffers from a severe pustular dermatosis, initially described as a pustular variant of psoriasis. We use DNA sequencing to refine the diagnosis of two of the children studied.

**Methods:**

Bidirectional Sanger sequencing was performed on the coding regions of the IL‐1Ra and IL‐36Ra genes (*IL1RN* and *IL36RN*, respectively), for the four affected children and their parents.

**Results:**

We identified a novel homozygous missense mutation in *IL36RN* in two siblings, and showed the molecular basis of the condition to be both distinct from psoriasis and distinct between the two families studied.

**Conclusions:**

We describe a novel mutation which underpins the diagnosis of childhood pustular dermatosis. Molecular diagnostics can be used to aid the clinical diagnosis and potential treatment of autoinflammatory conditions.

## Introduction

Autoinflammatory conditions are a group of genetic diseases, characterized by an innate immune response to endogenous cells, which can impact significantly on health and quality of life. The exact pathogenesis of autoinflammatory conditions is difficult to determine from their clinical phenotypes, which are frequently complex with overlapping features.^1^ The accurate identification of genetic mutations that underpin autoinflammatory conditions can help to determine their pathogenesis and thereby assist in determining the most appropriate treatment option.^2^

## Clinical presentation

### Family 1

**Figure 1:**
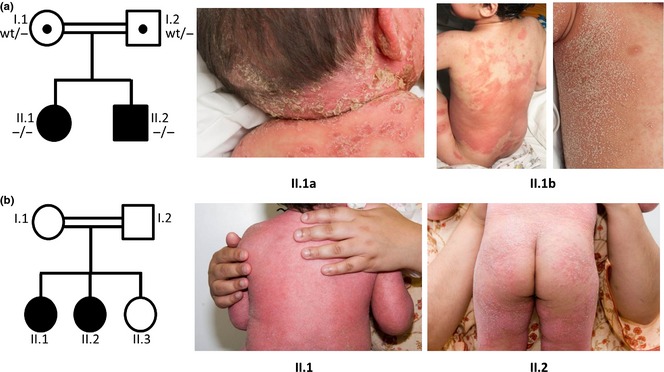
Family pedigrees and clinical photographs of affected children. (a) Family 1, with IL36RN mutation profile (*WT*, leucine amino acid; *‐*, proline amino acid), pictures show the girl's pustular dermatosis at age: 18 months (II.1a) and 2.5 years (II.1b). (b) Family 2, two elder daughters affected by pustular psoriasis.

An 18‐month‐old girl born to consanguineous Pakistani parents presented with a rapid onset of fever and systemic upset. Clinical examination revealed generalised erythema studded with follicular pustules concentrated mainly on the infant's limbs and trunk (Fig. 1a, II.1a). She had no pre‐existing skin condition. The sudden appearance of inflamed pustules and fever concurrent with clarithromycin use for an upper respiratory tract infection, suggested an initial diagnosis of acute generalised exanthematous pustulosis (*AGEP*; Table 1). Withdrawal of clarithromycin and the use of non‐steroidal topical treatment reduced the inflammation, supporting the initial diagnosis of AGEP. However, she presented with identical clinical features 12 months later (Fig. 1a, II.1b), this time in the absence of a potential exogenous cause. The diagnosis was reassigned to generalised pustular psoriasis (Table 1). Her 7‐year‐old brother also presented with a febrile illness, generalised erythema and pustules 2 months after her second episode of inflamed pustules. He was not receiving medication and had pre‐existing plaques of erythema and scaling on his limbs and chest. Skin biopsies from both children were consistent with a diagnosis of pustular psoriasis. Topical application of calcipotriol proved ineffective for both children. Systemic treatment with acitretin, 10 mg daily, for 6 months relieved the febrile symptoms and pustule formation of both siblings. The addition of methotrexate (5 mg for the girl, 7.5 mg for the boy), once a week, in combination with acitretin after the initial 6‐month period has improved the condition of both children.

**Table 1:** Disease characteristics

**1. Generalised Pustular Psoriasis (GPP)**
*Typical presentation*: Rapid development of widespread sterile pustules. Often associated with fever and fatigueMay have history of more typical Plaque Psoriasis. May be recurrent*Molecular basis*: Heterogeneous*Stimulus for presentation*: Mostly unknown, but may present in response to infection or withdrawal of steroid therapy**2. Acute generalised Exanthematous Pustulosis (AGEP)**
*Typical presentation*: Rapid development of widespread sterile pustules. Rarely associated with fever and systemic illness*Molecular basis*: Heterogeneous*Stimulus for presentation*: Most commonly exogenous agent use, e.g. antibiotics. Symptoms usually resolve within weeks of removal of causative stimulus**3. Deficiency of Interleukin‐36 Receptor Antagonist Disorder (DITRA)**
*Typical presentation*: Rapid development of widespread sterile pustules. Often associated with fever and fatigue*Molecular basis*: Mutations in *IL36RN*, which encodes the interleukin‐36 receptor antagonist cytokine*Stimulus for presentation*: Mostly unknown

### Family 2

The first‐born daughter of a consanguineous Pakistani couple developed an eczematous rash in the flexures and groin at 2 months of age. The rash recurred at 12 months and the girl presented at 18 months of age with exfoliative erythroderma and sparse hair. She was treated with topical steroids and oral flucloxacillin. She was lost to follow‐up, during which time she received oral prednisolone. She returned aged 23 months with fever, exfoliative erythroderma, palmoplantar pustules and Cushingoid features. During her recovery, psoriasiform lesions were noted on her lower back (Fig. 1b, II.1); Netherton's syndrome was excluded on skin biopsy, which showed normal Lympho‐Epithelial Kazal‐Type‐related Inhibitor (LEKTI) staining and features consistent with psoriasis. Over the ensuing 5 years she had recurrent eruptions of widespread migratory pustular psoriasis with fever and transient nail dystrophy, on a background of persistent flexural, vulval and generalised plaque psoriasis. Plaque and pustular lesions involved ~50% body surface area during her worst flares, necessitating several hospital admissions. Daily treatment with 10 mg acitretin was started at age 7 years. Her condition rapidly improved and although she has had several minor flares of plaque and pustular psoriasis since, she has not required admission and, aged 10 years, reports a good quality of life.

Her younger sister, now aged 6 years, followed a very similar disease course, developing severe exfoliative erythroderma with palmoplantar pustulosis aged 2 months (Fig. 1b, II.2), and progressing to frequent bouts of pustular psoriasis with systemic features and transient nail dystrophy. She has had scattered mild plaque psoriasis and improved significantly after daily treatment with 10 mg acitretin at age 3.5 years.

A third child, a daughter, was born in 2013. She has Down syndrome and, now at 18 months of age, has not shown any sign of skin disease.

## Genetics

interleukin (IL)‐1 superfamily contains 11 molecules that play a pivotal role in innate immunity, including proinflammatory cytokines IL‐1 (α and β) and IL‐36 (α, β and γ), and anti‐inflammatory receptor antagonist (Ra) cytokines IL‐1Ra and IL‐36Ra.^3^ Recently, genetics research has facilitated the sub‐classification of autoinflammatory pustular disorders caused by mutations in genes within the IL‐1 superfamily.^4^

Interleukin‐1Ra and IL‐36Ra are anti‐inflammatory cytokines that bind to the IL‐1 and IL‐36 cell membrane receptors, respectively, without initiating a downstream inflammatory response.^3^, ^5^ Interleukin‐1Ra and IL‐36Ra inhibit the binding of IL‐1 family proinflammatory cytokines to their membrane receptors, and hence act as inhibitors of IL‐1 and IL‐36 cytokine mediated inflammation. Recessively inherited null and missense mutations in the genes encoding IL‐1Ra (*IL1RN*) and IL‐36Ra (*IL36RN*) can cause the development of severe pustular dermatoses,^3^, ^5^, ^6^, ^7^ and are now recognized as discrete clinical diagnoses: deficiency of IL‐36Ra disorder (DITRA)^3^; and deficiency of IL‐1Ra disorder (DIRA).^5^

**Figure 2:**
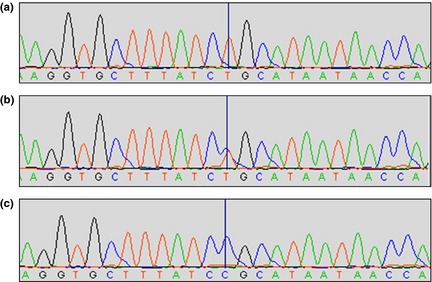
Staden display of *IL36RN* sanger sequencing results from Family 1. [vertical line shows cDNA position 62 at codon 21 in the *NM_173170.1* transcript]. (a) control DNA with a homozygous T/T genotype. (b) example of parent DNA with T/C heterozygous genotype. (c) example of affected child DNA with C/C homozygous genotype.

In this study, polymerase chain reaction and bidirectional Sanger sequencing were performed on DNA extracted from peripheral blood samples of the affected children and their consanguineous parents, to acquire a mutation profile of all known coding exons of *IL36RN* and *IL1RN*. A novel homozygous missense variant in *IL36RN,* c.62T>C p.Leu21Pro (NM_173170.1), present in an exon containing other disease‐causing mutations^3^ was found in both affected children of the first family, with a heterozygous genotype in their unaffected parents (Figs 1a and 2). No other homozygous or *de novo* variant was found exclusively in the affected children of the first family. Comparison with the 1000 genomes, Exome Variant Server and dbSNP databases identified that the c.62T>C variant has not been reported previously. *IL36RN* c.62T>C is also absent from the Manchester Centre for Genomic Medicine in‐house database, which contains whole‐exome sequencing data for more than 500 patients, of which ~25% are of Asian descent. Condel *in‐silico* prediction software^8^ provides a consensus score for popular missense prediction algorithms; Condel indicated that the p.Leu21Pro variant would have a deleterious impact on the structure of the *IL36RN* protein. The predicted disruption of the structure, and therefore function, of IL‐36Ra is consistent with the observed febrile illness and widespread pustular lesions in two of the four children reported in this study.^3^

In the absence of functional evidence, we cautiously define the *IL36RN* c.62T>C p.Leu21Pro variant as a ‘likely pathogenic’ mutation, and suggest the diagnosis in family 1 be redefined to DITRA.^3^ c.62T>C adds to an emerging array of mutations in *IL36RN* underpinning autoinflammatory disorders,^7^ and has implications for future treatment options for patients reported with this mutation.^2^ Initial evidence suggests that anakinra (Kineret^®^, Sobi, Inc, Waltham, MA, USA), a human recombinant IL‐1Ra, can be used to treat paediatric pustular dermatoses caused by mutations in *IL36RN*.^9^ Thus, transition of the children in the first family reported here to anakinra might be appropriate if their disease flares in the future. The monogenic nature of DITRA also has informed the genetic counselling that the family will receive, and will allow accurate and comprehensive parent education about the risk of additional offspring developing the disorder.

In contrast, homozygous or *de novo* mutations in *IL1RN* or *IL36RN* were not found in the affected children of the second family. The lack of mutation in these individuals indicates that despite the increasing resolution with which genetic technology can define autoinflammatory disease, complex heterogeneity remains a significant difficulty for the diagnosis of patients with overlapping clinical features.

In conclusion, genetic analysis of four Pakistani children suffering from pustular dermatoses uncovered a novel mutation in the IL‐1 family receptor antagonist gene *IL36RN*. The key benefits of understanding the molecular basis of the pustular dermatoses described here are an accurate assessment of: (i) the initial clinical diagnosis; (ii) the likelihood that additional offspring will inherit the disorder; and (iii) the most appropriate targeted therapy.
